# Implementation of Intraoperative Cone-Beam CT (Loop-X) in an Established Robotic-Assisted Pedicle Screw Program: An Epoch-Based Cohort Study

**DOI:** 10.3390/jcm15103749

**Published:** 2026-05-13

**Authors:** Julien N. Jost, Kristina Catalano, Jaqueline Lattmann, Debora Cipriani, Thomas Rhomberg, Lukas Andereggen, Gerrit A. Schubert, Markus Bruder

**Affiliations:** 1 Department of Neurosurgery, Cantonal Hospital Aarau, 5001 Aarau, Switzerland; 2 Faculty of Medicine, University of Bern, 3012 Bern, Switzerland; 3 Department of Neurosurgery, RWTH Aachen University, 52074 Aachen, Germany; 4 Department of Neurosurgery, University Hospital Frankfurt, 60629 Frankfurt, Germany

**Keywords:** robot-assisted spine surgery, pedicle screw fixation, intraoperative imaging, cone-beam CT, spinal navigation, implementation study

## Abstract

**Background/Objectives**: Reliable intraoperative imaging is essential for robotic-assisted (RA) pedicle screw placement. Mobile intraoperative cone-beam CT (iCBCT) systems like Loop-X have been introduced into RA workflows, but implementation data remain limited. We evaluated whether Loop-X introduction was associated with efficiency or safety, and whether associations differed by surgeon experience. **Methods**: We performed a retrospective epoch-based cohort study of 146 patients undergoing RA pedicle screw placement using 3D C-arm navigation (3D-BV) or Loop-X iCBCT. Outcomes were operating room (OR) time, estimated blood loss (EBL), and length of stay (LOS). The safety endpoint was 30-day complications (Clavien–Dindo grade III or higher). Multivariable regression models adjusted for patient- and procedure-related covariates; the complication model was kept parsimonious because of the limited number of events. Interaction terms tested surgeon experience. **Results**: After adjustment, implementation phase was not independently associated with OR time (β 30.76 min, 95% CI −31.97 to 93.48; *p* = 0.33), EBL (β −19.94 mL, 95% CI −287.01 to 247.14; *p* = 0.88), or LOS (β 3.21 days, 95% CI −3.31 to 9.72; *p* = 0.33). No significant interaction with surgeon experience was detected. Major complications occurred in 16 of 146 cases (11.0%) and were not associated with Loop-X implementation (OR 0.41, 95% CI 0.10–1.74; *p* = 0.224). Patient factors and procedural complexity were the main determinants of outcomes. **Conclusions**: In this cohort, Loop-X implementation in an RA pedicle screw program was not associated with deterioration in perioperative efficiency or short-term safety after adjustment. These findings support feasibility in practice, but do not establish superiority, equivalence, or imaging-specific effects.

## 1. Introduction

Robotic-assisted (RA) pedicle screw placement has become increasingly established in spine surgery, offering high accuracy and reproducibility across a wide range of indications [1]. Reliable intraoperative imaging is central to these workflows, as it provides the reference dataset for navigation and RA execution of instrumentation and thereby supports safe and efficient screw placement [2,3]. 

Fluoroscopy-based 3D C-arm navigation (3D-BV) has traditionally been widely used and is well integrated into many RA spine surgery programs. In parallel, intraoperative cone-beam CT (iCBCT) systems have emerged as an alternative imaging modality for navigated and RA spinal instrumentation [4,5,6]. Among these, the mobile Loop-X platform (Brainlab, Munich, Germany) combines iCBCT image acquisition with automated positioning and integration into contemporary navigation ecosystems [3,7,8].

Most published studies on iCBCT in spine surgery have focused on pedicle screw accuracy, revision rates, or radiation-related aspects [2,9,10]. By contrast, less attention has been given to the practical question of how a new imaging platform can be introduced into an already established RA workflow in routine clinical practice. This issue is clinically relevant because implementation of new intraoperative technologies extends beyond technical accuracy alone and may affect perioperative efficiency, safety, and usability across surgeons with different levels of experience.

In particular, it remains unclear whether sequential implementation of iCBCT within an established RA program is associated with changes in operating room (OR) performance, estimated blood loss (EBL), length of stay (LOS), or major complications during the transition phase. It is also uncertain whether the adoption of a new imaging platform may differentially affect junior and senior surgeons, which is especially relevant in teaching environments.

The aim of this study was therefore to evaluate the sequential implementation of Loop-X within an already established RA pedicle screw workflow in routine clinical practice. Specifically, we assessed whether this implementation phase was associated with changes in perioperative efficiency and major complications, and whether any association differed across surgeon experience levels. Given the epoch-based design, this study was intended as an implementation analysis rather than a contemporaneous head-to-head comparison of imaging modalities.

## 2. Materials and Methods

### 2.1. Study Design and Patient Population

This retrospective single-center cohort study included 146 consecutive patients who underwent RA pedicle screw placement at a tertiary spine surgery center. All procedures were performed as part of routine clinical care using a robotic guidance system (CIRQ, Version 2.0, Brainlab, Munich, Germany) [11,12]. The study period comprised two consecutive implementation phases defined by the intraoperative imaging modality used for robotic navigation: 3D-BV (Ziehm Vision RFD 3D, Version 6.073, Ziehm Imaging, Nuremberg, Germany) from May 2023 to December 2024 and iCBCT (Loop-X, Version 1.0, Brainlab, Munich, Germany) from January 2025 to January 2026.

There were no exclusions based on spinal region, surgical indication, or approach. The transition from 3D-BV to Loop-X was part of a continuous institutional workflow and did not coincide with intentional changes in surgical indication, staffing structure, or perioperative protocols. Because the two imaging platforms were used in consecutive rather than contemporaneous phases, imaging modality was analyzed as an epoch-based implementation exposure. Accordingly, the study was not designed to isolate a pure device effect independent of temporal, procedural, and team-related changes over time.

### 2.2. Surgical Technique and Imaging

All procedures were performed using RA pedicle screw placement according to standardized institutional protocols. Preoperative planning and intraoperative guidance were based on intraoperative imaging acquired either with 3D-BV or with the Loop-X system, a mobile robotic iCBCT platform providing CT-like 3D datasets for navigated spine surgery. Surgical approach (percutaneous vs. open), number of instrumented levels, and use of revision techniques were determined according to clinical indication and surgeon preference. Percutaneous approaches included both true percutaneous and transfascial techniques, whereas open screw placement involved detachment of the paravertebral musculature. Procedures were performed by surgeons with different levels of experience, classified as junior or senior according to institutional criteria. The cohort comprised cervical, thoracic, and lumbosacral procedures, reflecting routine clinical practice.

**Figure 1:**
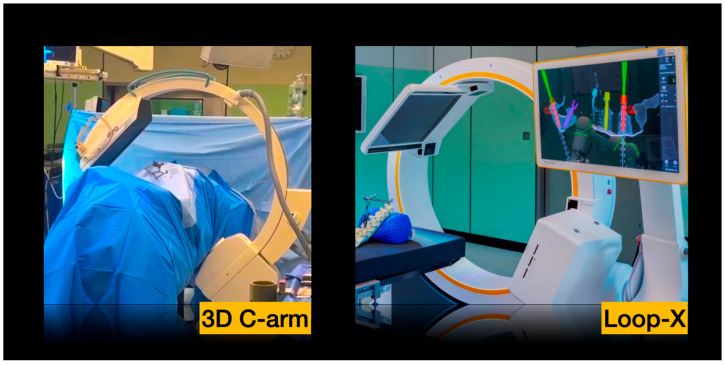
Imaging modalities used during the study period: 3D-BV and iCBCT (Loop-X). The images illustrate the two imaging platforms used for RA pedicle screw placement during the study period.

Representative images of both imaging platforms are shown in Figure 1 to illustrate the intraoperative imaging systems used during the two implementation phases.

### 2.3. Data Collection and Study Endpoints

Demographic data, comorbidities, procedural variables, and perioperative outcomes were extracted from electronic medical records. Baseline variables included age, sex, body mass index (BMI), American Society of Anesthesiologists (ASA) class, relevant comorbidities, revision surgery status, surgical approach, number of pedicle screws per case, and surgeon experience level. The primary perioperative outcomes were incision-to-closure OR time, EBL, and LOS. OR time was defined as the interval from skin incision to wound closure. EBL was recorded intraoperatively according to anesthesia documentation. LOS was defined as the number of postoperative days until discharge. Direct technical workflow metrics such as image acquisition time, registration time, registration failure, repeat scanning, technical troubleshooting, staff movement, and radiation exposure were not systematically recorded during the study period. The primary safety endpoint was the occurrence of major postoperative complications within 30 days, defined as Clavien–Dindo grade III or higher [13]. Complications were identified from inpatient records, postoperative encounters, and readmissions. Clavien–Dindo grades were harmonized before analysis, including alphanumeric grades such as IIIa, IIIb, IVa, and V. For descriptive reporting, major complications were categorized according to the dominant documented complication mechanism, including infection-related complications, screw-related revision or decompression, other surgical revisions, major medical complications or death, and iatrogenic visceral or vascular/plexus-related complications.

Screw-related safety outcomes were assessed using intraoperative three-dimensional imaging and/or postoperative CT. Radiographic screw position was evaluated according to the Gertzbein–Robbins classification [14]. Screw malposition was defined as a cortical breach greater than 2 mm, corresponding to Gertzbein–Robbins grade C or higher. Clinically relevant screw malposition was defined as radiographic malposition requiring postoperative screw revision or repositioning. Asymptomatic malpositioned screws were recorded separately when left in situ without revision. Intraoperative screw adjustments performed during the index procedure were considered part of the navigation-controlled placement workflow and were not classified as postoperative complications. New postoperative neurological deficits were recorded separately and were considered screw-related only when this relationship was supported by clinical and radiographic documentation.

Secondary outcomes included the interaction between implementation phase and surgeon experience for the perioperative outcomes, sensitivity analyses addressing early implementation and surgical approach, and descriptive technical safety indicators. Screw-related indicators included radiographic screw malposition according to the Gertzbein–Robbins classification, asymptomatic screw malposition managed without postoperative revision, postoperative screw revision or repositioning, and screw-related neurological deficits.

### 2.4. Surgeon Experience

Surgeon experience was categorized as junior or senior. Senior surgeons were board-certified neurosurgeons with a subspecialty focus on spine surgery, whereas junior surgeons were recently board-certified neurosurgeons in the early post-certification phase and operated under standard institutional supervision. Surgeon experience was analyzed as a potential effect modifier to explore whether associations between implementation phase and perioperative outcomes differed across experience levels.

### 2.5. Statistical Analysis

Continuous variables are presented as mean ± standard deviation or median with interquartile range, as appropriate; categorical variables were presented as counts and percentages. Distributional characteristics were assessed visually and descriptively before model fitting. Group comparisons between implementation phases were performed using Student’s *t* test or the Mann–Whitney U test for continuous variables and the chi-square test or Fisher’s exact test for categorical variables, as appropriate.

Because the two imaging modalities were used in consecutive epochs rather than contemporaneously, all analyses were interpreted as implementation-phase analyses rather than causal comparisons of imaging platforms. To address temporal and procedural confounding, chronological case number was included as a covariate to account for workflow maturation over time, and surgical approach was included because the proportion of percutaneous procedures differed substantially between phases. Additional procedural complexity was addressed by including revision surgery, interbody fusion/corpectomy, and the number of pedicle screws per case.

Multivariable regression models were used to assess the association between implementation phase and perioperative outcomes (OR time, EBL, LOS). Continuous outcomes were analyzed using general linear models including implementation phase, surgeon experience, and their interaction, adjusted for age, ASA class, surgical approach, revision surgery, interbody fusion/corpectomy, and number of pedicle screws. Model assumptions were assessed by inspection of residual plots. Because EBL and LOS may show skewed distributions in surgical cohorts, model results were interpreted together with non-parametric group comparisons and predefined sensitivity analyses.

Major complications, defined as Clavien–Dindo grade III or higher within 30 days, were analyzed using multivariable logistic regression. Given the limited number of events, the logistic regression model was restricted to a parsimonious set of clinically relevant covariates selected a priori. The primary logistic regression model included four predictors for 16 major complication events, corresponding to an events-per-variable ratio of 4. This low events-per-variable ratio was considered when interpreting the estimates, particularly the width of the confidence intervals. Potential collinearity between clinically related procedural variables was assessed descriptively before model fitting, and the final model was restricted to avoid simultaneous inclusion of highly overlapping covariates. Interaction terms were not included for this endpoint to reduce the risk of overfitting. Model stability was assessed by comparing the primary model with simplified sensitivity models, including models substituting diabetes mellitus or surgeon experience for selected covariates.

Prespecified sensitivity analyses were performed to evaluate the robustness of the perioperative outcome models. First, the first 10 Loop-X cases were excluded to assess whether early adoption influenced the results. Second, analyses were restricted to percutaneous procedures to reduce confounding by surgical approach. Propensity score-based approaches were considered; however, given the limited sample size, the strong coupling between implementation phase and surgical approach, and the risk of unstable estimates after matching or weighting, the primary strategy relied on covariate adjustment and restricted sensitivity analyses.

Missing data were handled using complete-case analysis because missingness was below 5% and no apparent pattern was observed. Effect estimates are reported with 95% confidence intervals. All tests were two-sided, and *p* < 0.05 was considered statistically significant. No adjustment for multiple comparisons was performed; secondary and sensitivity analyses were interpreted as exploratory. Analyses were conducted using IBM SPSS Statistics version 29.0 (IBM Corp., Armonk, NY, USA).

### 2.6. Ethics

Ethical approval was obtained from the Ethics Committee of Northwestern and Central Switzerland (Ethikkommission Nordwest und Zentralschweiz; approval number 2024-01216). This retrospective study analyzed existing medical data from patients who had provided general consent for the use of de-identified information for research purposes and was conducted in accordance with the Declaration of Helsinki and its later amendments.

## 3. Results

### 3.1. Patient Cohort and Baseline Characteristics

A total of 146 consecutive patients undergoing RA pedicle screw placement were included between May 2023 and January 2026. Of these, 76 cases were performed using 3D-BV and 70 using Loop-X following its implementation in January 2025. Overall, 1006 pedicle screws were placed. Baseline demographic characteristics were comparable between implementation phases. Mean age was 67 ± 15 years, and 55% of patients were male. BMI, ASA class, diabetes mellitus, cardiovascular disease, anticoagulation or antiplatelet therapy, and revision surgery status did not differ significantly between groups. The mean number of pedicle screws per case was 6.9 ± 3.4 overall, with no statistically significant difference between 3D-BV and Loop-X (7.3 ± 4.0 vs. 6.4 ± 2.5, *p* = 0.33). Percutaneous approaches were significantly more frequent during the Loop-X phase (93% vs. 54%, *p* < 0.001). The proportion of junior surgeons was higher in the Loop-X phase (56% vs. 39%, *p* = 0.050). Detailed baseline and procedural characteristics are shown in Table 1.

### 3.2. Perioperative Outcomes

#### 3.2.1. OR Time

**Figure 2:**
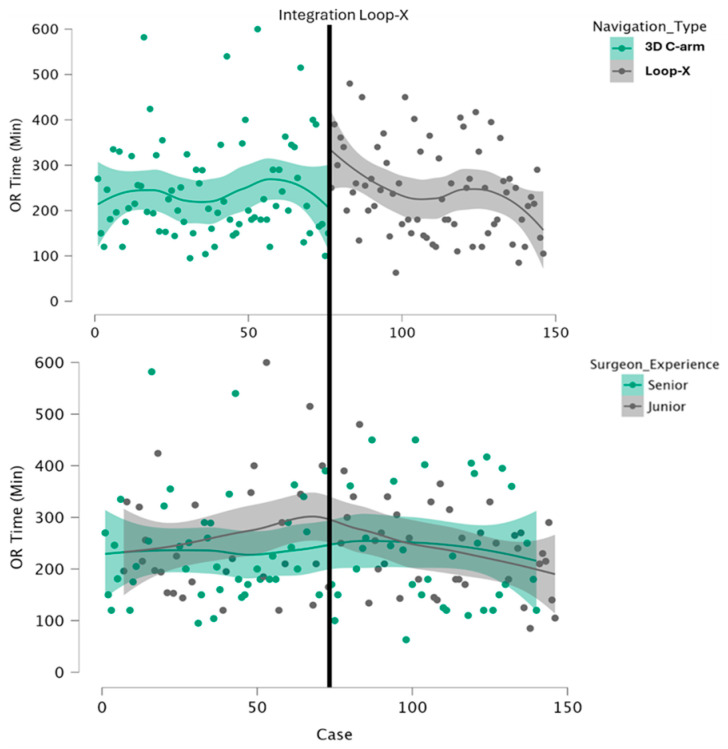
Incision-to-closure OR time across consecutive cases. Scatter plots with locally smoothed trends illustrate OR time across consecutive cases, stratified by imaging modality and surgeon experience. The figure is descriptive and is intended to visualize temporal workflow patterns during the study period. It does not represent a formal learning-curve analysis or causal assessment of imaging modality effects. Shaded areas indicate 95% confidence intervals.

Figure 2 illustrates incision-to-closure OR time across consecutive cases, stratified by implementation phase and surgeon experience. A transient increase in OR time was observed immediately after Loop-X implementation, followed by attenuation over subsequent cases. This descriptive pattern was not associated with a statistically detectable sustained increase in OR time. In the adjusted regression model, implementation phase was not independently associated with OR time (β 30.76 min, 95% CI −31.97 to 93.48; *p* = 0.33; Table 2). Surgeon experience was likewise not significantly associated with OR time (β 7.00 min, 95% CI −24.93 to 38.93; *p* = 0.67). No statistically detectable interaction between implementation phase and surgeon experience was observed for OR time. The strongest determinant of OR time was procedural extent; accordingly, cases without an additional anterior approach were significantly shorter.

#### 3.2.2. EBL

EBL did not differ significantly between implementation phases after multivariable adjustment (β −19.94 mL, 95% CI −287.01 to 247.14; *p* = 0.88; Table 2). Open surgery was independently associated with greater EBL compared with percutaneous surgery (β 244.59 mL, 95% CI 63.46 to 425.72; *p* = 0.008). Neither surgeon experience nor age or ASA class showed a statistically significant independent association with EBL in the primary model.

#### 3.2.3. LOS

Implementation phase was not independently associated with LOS in the adjusted model (β 3.21 days, 95% CI −3.31 to 9.72; *p* = 0.33; Table 2). Higher ASA class was associated with longer hospitalization (β 4.46 days, 95% CI 2.06 to 6.87; *p* < 0.001). Age showed a non-significant trend toward shorter LOS, whereas surgeon experience and implementation phase were not significant predictors.

### 3.3. Major Complications

Major complications (Clavien–Dindo grade III or higher) occurred in 16 of 146 cases (11%). In the primary multivariable logistic regression model, Loop-X implementation was not associated with major complications (OR 0.41, 95% CI 0.10–1.74; *p* = 0.224; Table 3). ASA class and number of screws were independently associated with major complications, whereas implementation phase and surgeon experience were not.

The most frequent major complication category was deep surgical site infection requiring intervention, followed by severe medical complications or death without a documented screw-related cause. Screw-related revision or decompression was documented in one case. Details of major complications are summarized in Table 4.

Sensitivity models yielded comparable findings. In a model substituting diabetes mellitus for anticoagulation, Loop-X remained unassociated with major complications (OR 0.49, 95% CI 0.12–2.09; *p* = 0.335), whereas diabetes mellitus was associated with increased odds of major complications (OR 6.27, 95% CI 1.63–24.06; *p* = 0.008). In a model including surgeon experience, implementation phase again remained non-significant (OR 0.49, 95% CI 0.12–1.91; *p* = 0.301), and surgeon experience was not independently associated with major complications.

### 3.4. Sensitivity Analyses for Perioperative Outcomes

Sensitivity analyses addressing early implementation effects and surgical approach are summarized in Table 5. Exclusion of the first 10 Loop-X cases did not materially alter the findings for OR time, EBL, or LOS. Likewise, restriction to percutaneous procedures only did not change the overall interpretation of the results. Across sensitivity analyses, no statistically detectable interaction between implementation phase and surgeon experience was observed for OR time or LOS. For EBL, the implementation phase × surgeon experience interaction reached statistical significance in the percutaneous-only analysis (*p* = 0.01). This finding should be interpreted cautiously because the analysis was exploratory, event-independent, based on a restricted subgroup, and potentially affected by residual confounding and small subgroup sizes.

### 3.5. Technical Safety Outcomes

Screw-related and clinical safety outcomes are summarized in Table 6. Among 1006 pedicle screws, 555 were placed during the 3D-BV phase and 451 during the Loop-X phase. Radiographic screw malposition, defined as Gertzbein–Robbins grade C or higher, was identified in 21 screws overall (2.1%). Radiographic malposition was observed in 16 of 555 screws during the 3D-BV phase and in 5 of 451 screws during the Loop-X phase (2.9% vs. 1.1%; *p* = 0.074).

Most malpositioned screws were asymptomatic and were managed without revision. Postoperative screw revision or repositioning was required for three screws, all during the 3D-BV phase (3/555, 0.5% vs. 0/451, 0.0%; *p* = 0.255). At the patient level, major complications occurred in 16 of 146 patients (11.0%), and new postoperative neurological deficits from all causes were documented in 13 patients overall. These deficits consisted of postoperative hip flexor weakness in the LLIF subgroup; most were self-limiting, whereas two persisted permanently, corresponding to M3 hip flexion strength [15]. No screw-related neurological deficit was observed.

## 4. Discussion

In this epoch-based retrospective cohort, sequential implementation of iCBCT (Loop-X) within an established RA pedicle screw program was not associated with a statistically detectable worsening of OR time, EBL, LOS, or major complication rates after adjustment for measured confounders.

These findings suggest that Loop-X could be incorporated into an existing RA workflow without a measurable deterioration in the available perioperative workflow proxies. However, because direct workflow components such as image acquisition time, registration time, repeat scanning, troubleshooting, staff movement, and radiation exposure were not systematically captured, the present data cannot determine which specific technical or organizational factors contributed to the observed stability.

Previous studies evaluating iCBCT in spine surgery have mainly focused on technical endpoints such as pedicle screw accuracy, revision rates, or radiation-related parameters [3,9,12,16]. By contrast, less is known about the real-world implementation of such systems within already established RA workflows. In this context, our findings add a pragmatic perspective by suggesting that sequential adoption of Loop-X did not coincide with a clear deterioration in short-term perioperative performance or clinical safety.

### 4.1. Integration of Loop-X into an Established Robotic Workflow

Introducing a new imaging modality into a RA spine unit represents a critical organizational and technical step, as it affects intraoperative logistics, team coordination, and time efficiency. In the present cohort, a transient descriptive increase in OR time was observed immediately after Loop-X implementation, followed by attenuation over subsequent cases. However, implementation phase was not independently associated with OR time after adjustment for measured confounders, including chronological case number, surgical approach, and procedural complexity. These findings suggest that Loop-X could be incorporated into an existing RA workflow without a measurable deterioration in the available perioperative workflow proxies.

Potential practical advantages of mobile iCBCT systems may have contributed to implementation feasibility. However, direct workflow components such as image acquisition time, registration success, repeat scanning, radiation exposure, staff movement, or technical troubleshooting were not formally measured; therefore, the present data cannot determine which specific technical or organizational factors contributed to workflow stability.

### 4.2. Surgeon Experience and Adoption

A further objective was to explore whether observed associations differed by surgeon experience [17,18,19,20]. We did not detect statistically significant interaction effects between implementation phase and surgeon experience for the main perioperative endpoints. This should be interpreted cautiously: the present sample was not powered to establish equivalence between experience groups, and the absence of a statistically significant interaction does not exclude smaller but potentially relevant differences. This finding is particularly relevant for teaching institutions and high-volume spine centers, where new technologies must be integrated not only by experienced surgeons but also within structured training environments. Our results suggest that Loop-X integration does not introduce an additional barrier for less experienced surgeons, supporting its applicability in multidisciplinary and educational settings.

While descriptive trends in OR time over consecutive cases were illustrated, this study was not designed for a formal learning curve analysis using CUSUM or time-series modeling. A dedicated assessment of user adaptation and intra-team learning dynamics is planned for a separate analysis.

### 4.3. Perioperative Stability During Implementation

Beyond OR time, EBL and LOS were not adversely associated with Loop-X implementation after adjustment. However, these outcomes are influenced by multiple factors beyond the imaging platform, including surgical approach, procedural complexity, patient comorbidity, and perioperative management. The marked increase in percutaneous procedures during the Loop-X phase is particularly important and may have influenced EBL independently of the imaging modality. Therefore, any observed differences in EBL or recovery should not be interpreted as device-specific effects.

To address potential sources of bias, sensitivity analyses were performed. Exclusion of the first 10 Loop-X cases yielded results consistent with the primary analyses, arguing against a relevant learning-curve or early adoption effect. Restriction to percutaneous procedures likewise did not materially change the overall interpretation, although residual confounding by case selection and procedural complexity remains possible. Additional analyses incorporating procedural complexity proxies, including the number of pedicle screws and interbody fusion/corpectomies, confirmed that workflow and perioperative outcomes were primarily driven by case complexity and patient-related factors rather than imaging modality or surgeon experience.

Taken together, these sensitivity analyses support the robustness of the primary results and suggest that the observed findings reflect stable workflow integration rather than temporal, procedural, or experience-related confounding.

Screw-related safety was assessed using standardized Gertzbein–Robbins grading, allowing radiographic screw malposition to be reported separately from clinically relevant screw revision. Radiographic malposition was uncommon in both implementation phases, and postoperative screw revision was required for three screws, all during the 3D-BV phase. Importantly, none of the revised screws was associated with a screw-related neurological deficit. These findings provide additional reassurance that implementation of Loop-X was not accompanied by an apparent screw-related safety signal. Nevertheless, screw-level comparisons should be interpreted cautiously because screws are clustered within patients and procedures, and the study was not primarily designed or powered as a formal radiographic accuracy comparison.

### 4.4. Clinical and Organizational Implications

From a practical standpoint, our findings suggest that Loop-X can be integrated into RA spine surgery programs without measurable deterioration in the available perioperative workflow proxies. The observed consistency across surgeon experience levels supports the feasibility of integration in a teaching environment, although subgroup analyses were underpowered.

These results may inform institutional decision-making when considering the adoption of mobile iCBCT systems such as Loop-X in conjunction with RA platforms, particularly in environments prioritizing standardization, efficiency, and training.

### 4.5. Radiation Exposure Considerations

Although not directly assessed in this study, radiation exposure remains an important factor when comparing intraoperative imaging modalities. Previous studies have shown that iCBCT systems can be associated with higher or comparable radiation doses depending on protocol and user experience [5]. Future studies evaluating Loop-X integration should include quantitative dosimetry and exposure metrics to guide safety assessments and institutional policy.

### 4.6. Limitations

This study has important limitations. First, the retrospective epoch-based design introduces substantial potential for temporal confounding. The two imaging modalities were used in consecutive rather than contemporaneous periods; therefore, the observed findings may reflect not only the imaging platform but also workflow maturation, changes in case selection, increasing team experience, and evolving perioperative practice. Although chronological case number was included as a covariate and sensitivity analyses were performed, residual confounding cannot be excluded. In addition, the two implementation phases differed in duration and case density. The 3D-BV phase covered approximately 20 months, whereas the Loop-X phase covered approximately 12 months, with a similar number of cases performed during the shorter Loop-X epoch. This asymmetric case accrual may reflect temporal and institutional changes, including increasing procedural volume, team familiarity, referral patterns, or case selection, and represents an additional source of residual confounding that cannot be fully separated from the implementation effect.

Second, the marked increase in percutaneous procedures during the Loop-X phase represents a major source of confounding, particularly for EBL and potentially also for OR time and postoperative recovery. Restriction to percutaneous procedures was performed as a sensitivity analysis to reduce this bias, but this approach cannot fully account for differences in indication, complexity, or surgical strategy between epochs.

Third, the study relied on indirect perioperative proxies of implementation rather than direct workflow metrics such as image acquisition time, registration success, repeat scanning, radiation exposure, technical troubleshooting, or staffing requirements. Therefore, the study cannot determine which specific technical components of the Loop-X workflow contributed to implementation feasibility or whether radiation exposure differed between imaging platforms.

Fourth, the number of major complications was limited. The primary logistic regression model included 16 events and four predictors, corresponding to an events-per-variable ratio of 4; therefore, the resulting odds ratios and wide confidence intervals should be interpreted cautiously. Accordingly, the logistic regression model was intentionally parsimonious, interaction terms were avoided for this endpoint, and the results should be interpreted as exploratory rather than definitive.

Fifth, although screw position was assessed using Gertzbein–Robbins grading, screw-level outcomes were not the primary endpoint of this study and screws are clustered within patients and procedures. Therefore, screw-related analyses should be interpreted as descriptive safety indicators rather than as a definitive accuracy comparison between imaging modalities.

Finally, one author reports consultancy for Brainlab AG, the manufacturer of the robotic and Loop-X systems used in this study. This potential conflict reinforces the need for cautious interpretation. Accordingly, the conclusions are deliberately limited to implementation feasibility and do not claim superiority, equivalence, or imaging-specific benefit.

## 5. Conclusions

In this retrospective epoch-based real-world cohort, implementation of iCBCT (Loop-X) in an established RA pedicle screw program was not associated with a clear deterioration in perioperative efficiency, major complications, or screw-related safety indicators after adjustment for measured confounders. These findings support implementation feasibility in routine practice, but they should not be interpreted as evidence of superiority, equivalence, or imaging-specific benefit independent of temporal and procedural confounding. Future prospective studies should include contemporaneous comparison groups, direct workflow metrics, radiation exposure, and standardized screw accuracy endpoints.

## Figures and Tables

**Table 1:** Baseline patient and procedural characteristics by implementation phase.

Variable	Overall (*n* = 146)	3D-BV (*n* = 76)	Loop-X (*n* = 70)	*p* Value
Age (years)	67 ± 15	67 ± 16	67 ± 14	0.59
Male sex, *n* (%)	80 (55%)	42 (55%)	38 (54%)	0.91
BMI (kg/m^2^)	27.3 ± 5.3	27.5 ± 5.6	27.0 ± 5.1	0.61
ASA score median [IQR]	3.0 [2.0–3.0]	3.0 [2.0–3.0]	3.0 [2.0–3.0]	0.22
Diabetes mellitus, *n* (%)	32 (22%)	20 (26%)	12 (17%)	0.18
Cardiovascular disease, *n* (%)	88 (60%)	50 (66%)	38 (54.3%)	0.16
Anticoagulation/antiplatelet therapy, *n* (%)	49 (34%)	29 (38%)	20 (29%)	0.2
Revision surgery, *n* (%)	38 (26%)	22 (29%)	16 (23%)	0.40
Percutaneous approach, *n* (%)	106 (73%)	41 (54%)	65 (93%)	<0.001
Number of pedicle screws per case	6.9 ± 3.4	7.3 ± 4.0	6.4 ± 2.5	0.33
Junior surgeon, *n* (%)	69 (47%)	30 (39%)	39 (56%)	0.05

**Table 2:** Multivariable regression models for perioperative outcomes.

Predictor	OR Time, β (95% CI)	*p* Value	EBL, β (95% CI)	*p* Value	LOS, β (95% CI)	*p* Value
Age (per year)	0.37 (−0.68 to 1.41)	0.49	−2.86 (−7.30 to 1.59)	0.21	−0.09 (−0.20 to 0.02)	0.09
ASA (per class)	7.20 (−15.98 to 30.38)	0.54	64.46 (−34.23 to 163.15)	0.20	4.46 (2.06 to 6.87)	<0.001
Case number	−0.32 (−1.09 to 0.46)	0.42	−1.84 (−5.14 to 1.46)	0.27	−0.06 (−0.14 to 0.02)	0.14
Implementation phase (Loop-X vs. 3D-BV)	30.76 (−31.97 to 93.48)	0.33	−19.94 (−287.01 to 247.14)	0.88	3.21 (−3.31 to 9.72)	0.33
Open approach (vs. percutaneous)	41.50 (−1.04 to 84.04)	0.056	244.59 (63.46 to 425.72)	0.008	−0.26 (−4.68 to 4.16)	0.91
No additional anterior approach	−98.18 (−130.99 to −65.37)	<0.001	−99.82 (−239.52 to 39.89)	0.16	1.73 (−1.68 to 5.14)	0.32
Junior surgeon	7.00 (−24.93 to 38.93)	0.67	91.47 (−44.49 to 227.43)	0.19	0.49 (−2.83 to 3.80)	0.77

**Table 3:** Multivariable logistic regression model for major complications (Clavien–Dindo grade ≥ III).

Effect	OR	95% CI	*p* Value
Loop-X vs. 3D-BV	0.41	0.10–1.74	0.22
Junior vs. Senior	2.33	0.49–11.12	0.29
ASA	8.79	3.12–24.78	<0.001
Number of screws	1.29	1.07–1.55	0.01

**Table 4:** Details of major complications within 30 days.

Complication Category	Overall *n* = 16	3D-BV *n* = 11	Loop-X *n* = 5
Deep surgical site infection requiring surgical or interventional treatment	8	5	3
Screw-related revision surgery or decompression	1	1	0
Non-infectious surgical revision or implant-related reoperation	2	2	0
Severe medical complication or death not attributed to screw malposition	3	2	1
Approach-related visceral, vascular, or plexus complication	2	1	1
Total	16	11	5

Major complications were defined as Clavien–Dindo grade III or higher within 30 days. Complications were categorized according to the dominant documented mechanism leading to intervention, readmission, or death. In cases with overlapping features, classification was based on the primary clinically documented reason for treatment escalation.

**Table 5:** Sensitivity analyses assessing robustness of workflow outcomes.

Outcome	Sensitivity Analysis	Imaging Modality *p*	Surgeon Experience *p*	Imaging Modality × Experience *p*	Significant Covariates
OR time	Excluding first 10 Loop-X cases	0.89	0.75	0.41	Number of pedicle screws (<0.001)
OR time	Percutaneous-only	0.51	0.42	0.37	Number of pedicle screws (0.003)
EBL	Excluding first 10 Loop-X cases	0.06	0.30	0.07	Percutaneous approach (0.038), number of pedicle screws (<0.001)
EBL	Percutaneous-only	0.07	0.06	0.01	—
LOS	Excluding first 10 Loop-X cases	0.61	0.90	0.85	ASA (0.002), age (0.048), number of pedicle screws (0.007)

Note: The dash indicates that no additional covariate reached statistical significance in this sensitivity model apart from the reported implementation phase × surgeon experience interaction.

**Table 6:** Descriptive technical safety outcomes.

Outcome	Overall	3D-BV	Loop-X	*p* Value
Total number of pedicle screws	1006	555	451	—
Radiographic screw malposition, *n*/N screws (%)	21/1006 (2.1%)	16/555 (2.9%)	5/451 (1.1%)	0.074
Asymptomatic screw malposition managed without postoperative revision, *n*/N screws (%)	18/1006 (1.8%)	13/555 (2.3%)	5/451 (1.1%)	0.176
Postoperative screw revision/repositioning, *n*/N screws (%)	3/1006 (0.3%)	3/555 (0.5%)	0/451 (0.0%)	0.255
Screw-related neurological deficit, *n*/N patients (%)	0/146 (0.0%)	0/76 (0.0%)	0/70 (0.0%)	—
New postoperative neurological deficit, all causes, *n*/N patients (%)	13/146 (8.9%)	5/76 (6.6%)	8/70 (11.4%)	0.388
Major complications, Clavien–Dindo ≥ III, *n*/N patients (%)	16/146 (11.0%)	11/76 (14.5%)	5/70 (7.1%)	0.191

## Data Availability

The datasets generated and/or analyzed during the current study are not publicly available due to institutional and data protection restrictions but are available from the corresponding author on reasonable request and with permission of the responsible ethics authority/institution.

## Abbreviations

The following abbreviations are used in this manuscript:

RA	robotic-assisted
iCBCT	intraoperative cone-beam computed tomography
3D-BV	fluoroscopy-based 3D C-arm navigation
OR	operating room
EBL	estimated blood loss
LOS	length of stay
